# Exploring the cockatiel (*Nymphicus hollandicus*) fecal microbiome, bacterial inhabitants of a worldwide pet

**DOI:** 10.7717/peerj.2837

**Published:** 2016-12-22

**Authors:** Luis David Alcaraz, Apolinar M. Hernández, Mariana Peimbert

**Affiliations:** 1 Laboratorio Nacional de Ciencias de la Sostenibilidad, Instituto de Ecología, Universidad Nacional Autonóma de México, Mexico City, Mexico; 2 Departamento de Ciencias Naturales, Unidad Cuajimalpa, Universidad Autónoma Metropolitana, Mexico City, Mexico

**Keywords:** Cockatiel, Microbiome, Erysipelotrichaceae, Comparative bird microbiome

## Abstract

**Background:**

Cockatiels (*Nymphicus hollandicus*) were originally endemic to Australia; now they are popular pets with a global distribution. It is now possible to conduct detailed molecular studies on cultivable and uncultivable bacteria that are part of the intestinal microbiome of healthy animals. These studies show that bacteria are an essential part of the metabolic capacity of animals. There are few studies on bird microbiomes and, to the best of our knowledge, this is the first report on the cockatiel microbiome.

**Methods:**

In this paper, we analyzed the gut microbiome from fecal samples of three healthy adult cockatiels by massive sequencing of the 16S rRNA gene. Additionally, we compared the cockatiel fecal microbiomes with those of other bird species, including poultry and wild birds.

**Results:**

The vast majority of the bacteria found in cockatiels were *Firmicutes*, while *Proteobacteria* and *Bacteroidetes* were poorly represented. A total of 19,280 different OTUs were detected, of which 8,072 belonged to the *Erysipelotrichaceae* family.

**Discussion:**

It is relevant to study cockatiel the microbiomes of cockatiels owing to their wide geographic distribution and close human contact. This study serves as a reference for cockatiel bacterial diversity. Despite the large OTU numbers, the diversity is not even and is dominated by *Firmicutes* of the *Erysipelotrichaceae* family. Cockatiels and other wild birds are almost depleted of *Bacteroidetes*, which happen to be abundant in poultry-related birds, and this is probably associated with the intensive human manipulation of poultry bird diets. Some probable pathogenic bacteria, such as *Clostridium* and *Serratia,* appeared to be frequent inhabitants of the fecal microbiome of cockatiels, whereas other potential pathogens were not detected.

## Introduction

The study of microbiology has been revolutionized by the development of more efficient and cheaper DNA sequencing techniques; this has allowed the study of communities without the need to cultivate and isolate each colony. These technological developments have established that a healthy human contains 10–100 trillion bacterial cells (Turnbaugh et al., 2007); recent advances in the estimation of bacterial to human cell numbers suggest a 1:1 ratio of bacteria: human cells, including erythrocytes in the estimation (Sender, Fuchs & Milo, 2016). The above information provided motivation to reanalyze the microorganisms associated with animals, providing a very different view of what we consider a healthy or a sick state.

There are relatively few studies on the gut microbial diversity of birds using next generation sequencing (Waite & Taylor, 2014; Hird et al., 2015; Lewis, Moore & Wang, 2016). The avian microbiome models that have been extensively studied are chickens and turkeys, and avian poultry microbiome studies have focused mainly on improving the health and weight gain of birds without using antibiotics, as in mammals the presence of *Lactobacillus* has been of particular relevance (Stanley, Hughes & Moore, 2014; Danzeisen et al., 2015). Studies on the microbiome of vultures (*Coragyps atratus*), which are animals that feed on decomposing meat rich in toxins, and also on that of penguins (multiple penguin species: *Aptenodytes patagonicus*, *Pygoscelis papua, Eudyptes chrysolophus, Eudyptula minor*), show an increased abundance of *Fusobacteria* (Dewar et al., 2013; Roggenbuck et al., 2014). The microbes of kakapos, parrots endemic to New Zealand that are critically endangered, have been studied as part of their conservation program (Waite & Taylor, 2014). In addition, there are studies of some other bird species such as the Hoatzin that have microbiota similar to the rumen of cows *(Bos taurus*), which is explained by their forage-based diet (Wright, Northwood & Obispo, 2009).

Both in the wild and in captivity, cockatiels (*Nymphicus hollandicus*) feed primarily on seeds, but they also eat fruits and vegetables. Like all psittacines (parrots), they are characterized by not having ceca, which has been attributed to a low-fiber diet (DeGolier, Mahoney & Duke, 1999). Cockatiels are gregarious, small, elegantly colorful and their reproduction in captivity is relatively simple, making them a good choice as a pet. Cockatiel is the only member from the family Cacatuidae; these birds are naturally distributed in Australia, with a global distribution as a pet and ornamental bird. *N. hollandicus* shows social behaviors; in the wild, they are grouped in flocks of 27 birds on average. However, when there is a shortage of food, flocks increase their size up to 100 birds (Jones, 1987).

There are some studies on the cultivable bacteria of cockatiels, but very few of those were performed on healthy birds. A previous report on cockatiel microbes studied the bacterial diversity in their skin, from which 37 colonies were isolated, 18 colonies corresponding to *Staphylococcus* and 5 to *Corynebacterium* (Lamb et al., 2014). This is the first report of the cockatiels fecal microbiomes at the preprint publication (Alcaraz, Hernandez & Peimbert, 2016), while the manuscript was under peer review another group published results of fecal microbiomes for bird pets (Garcia-Mazcorro et al., 2016). Given the wide distribution of cockatiels as pets, it is important to study the biodiversity of bacteria associated with these birds. In this paper, we describe the fecal microbial diversity of healthy adult cockatiels using next generation sequencing and analysis of the ribosomal 16S gene; additionally, cockatiel diversity is compared with that observed in other predominant granivorous birds.

## Methods

### Sampling

Fecal samples from three healthy adult cockatiels (*Nymphicus hollandicus*) were obtained from two commercial breeders at the Sonora Market in Mexico City: cockatiel 1 was bred by “Local 2”; cockatiels 2 and 3 by “El Refugio” the specimens were healthy and lived in captivity. The first fecal deposition of the day was immediately collected with cotton swabs, and the samples were stored at −80 °C in resuspension buffer (50 mM NaCl, 10 mM Tris–HCl pH 7.5, 10 mM EDTA) until processed. No special permission was required for this work; the bird sellers gave us permission to sample stool, and no birds were harmed in this study. *Nymphicus hollandicus* is listed as “Least Concern ver 3.1” in the Red List of the International Union for Conservation of Nature (IUCN; http://www.iucnredlist.org/details/22684828/0).

### DNA preparation

DNA extraction was originally done with standard commercial kits but they showed poor performance for cockatiel feces samples compared to standard lysis and DNA purification methods used in this work (Sambrook, Fritsch & Maniatis, 1989). For each sample, 30 µl was resuspended in 150 µl of GTE buffer (50 mM glucose, 10 mM EDTA, 25 mM Tris–HCl pH 8.0). Cell lysis was achieved by incubation with 0.1 mg/mL of lysozyme for 5 min at 25 °C, and then SDS was added to a final concentration of 2%. Two hundred microliters of phenol were added, and the solution was incubated for 15 min at 55 °C. The aqueous phase was separated and re-extracted with phenol-chloroform-isoamyl alcohol (25:24:1), and then with chloroform-isoamyl alcohol (24:1). DNA was precipitated with sodium acetate and ethanol at −20 °C and resuspended in water. Later, binding buffer, which includes guanidine-HCl and proteinase K, was added; the DNA was bound to a spin column of silica gel, the column was washed two times, and finally, the DNA was eluted in 50 µl water. In summary, DNA purification was performed by standard procedures (Sambrook, Fritsch & Maniatis, 1989), except that phenol extraction was performed at 55 °C. The DNA was further purified by use of a High Pure PCR Template Kit (Roche Diagnostics GmbH, Mannheim, Germany) according to manufacturer’s instructions. DNA isolation method modifications are common practice; the method used by the Human Microbiome Project was originally intended for DNA purification from soil. Sambrook protocol is optimized for Gram-negative bacteria, in our results we observe dominance for Firmicutes (91%) which means that the method worked fine for Gram-positive bacteria too, which are hard to recover because lysis efficiency.

### Amplification

Three PCRs were performed for each sample. The primers MiSeq341F (5′-TCGTCGGCAGCGTCAGATGTGTATAAGAGACAGCCTACGGGNGGCWGCAG-3′) and MiSeq805R (5′-GTCTCGTGGGCTCGGAGATGTGTATAAGAGACAGGACTACHV GGGTATCTAATCC-3′) were used. The 3′ ends of the primers amplify regions V3 and V4 of the 16S gene (Herlemann et al., 2011), while the 5′ ends are Illumina^®^ adapter sequences for MiSeq™ (Illumina, San Diego, CA, USA). Three independent PCR reactions were performed for each sample and then pooled before sequencing. PCR were carried out in a final volume of 20 µl containing 250 µM dNTPs, 0.5 µM of each primer, 0.02 U Taq Platinum (Invitrogen, Carlsbad, CA, USA), and 10X Taq Platinum buffer containing 1.5 mM MgCl_2_. The protocol used for PCR reactions was as follows: initial denaturation at 95 °C for 3 min, followed by 25 cycles consisting of denaturation at 94 °C for 30 s, annealing at 55 °C for 30 s and extension at 72 °C for 1 min, and a final extension step at 72 °C for 5 min. PCR products were purified with a High Pure PCR Product Purification Kit (Roche Diagnostics GmbH, Mannheim, Germany).

### Sequencing

We used the National Autonomous University of Mexico’s Massive DNA Sequencing Facility UUSMD services to build sequencing libraries and MiSeq™ 300 bp paired ends, following directions from Illumina^®^ (Illumina, San Diego, CA, USA).

### Data analysis

Raw reads were processed and quality filtered using FASTQC (http://www.bioinformatics.babraham.ac.uk/projects/fastqc/), and Fastx-Toolkit (http://hannonlab.cshl.edu/fastx_toolkit/). The reads were assembled and merged, selecting a minimum length of 470 bp, minimum overlap of 15 bp and a quality cut-off for the assembly of 0.95, removing any ambiguous bases. The minimum length of 470 bp includes adapter sequences for MiSeq; sequences shorter than 470 bp were not paired. The algorithm used for merging paired-end sequences was PANDASEQ (Masella et al., 2012), which uses a full read length (-O parameter) by default. The adjusted parameter was (-o), which is defined as the minimum overlap; the default for -o is 1, so we are 15 bases higher than the default. Raising the -o number does not increase the number of merged sequences because if the overlap is too short, the sequence will score poorly, and it will be discarded by the −*t* parameter, the threshold of which ranges from 0–1 with a default of 0.6; we used a value of 0.95, which takes into account the alignment score of two matching sequences with the very same pair-end identifier. Physical DNA chimeras are typically formed during the PCR step, but stringency of merging parameters affects the likelihood that paired-end reads will result in new computer generated chimeras. Operational taxonomic units (OTUs) were picked with cd-hit-est using a 97% identity cut-off. OTU representative sequences were selected with *pick_rep_set.py* script from the QIIME pipeline (Caporaso et al., 2010). Taxonomic assignment of the representative OTUs was performed using BLAST (e-value = 1e−10) against Greengenes DB (v 13.8; DeSantis et al., 2006). Chimeras were identified by ChimeraSlayer (Haas et al., 2011). Chimeras, mitochondrial sequences and chloroplast sequences were removed after taxonomy assignment by string extraction. All statistical and diversity analyses were performed in R: phyloseq package (McMurdie & Holmes, 2013); plots were performed using the ggplot2 package and RColorBrewer library palettes. Diversity was also calculated by the R package breakaway (Willis, Bunge & Whitman, 2015) and the CatchAll program (Bunge et al., 2012). Species assignments were done using Greengenes DB (v13.8; DeSantis et al., 2006), with an e-value lower than e−100, then in a second round the positive sequences were taxonomic assigned by RDP naïve Bayesian classifier (Qiong et al., 2007), we assigned *Clostridium colinum* with a mean probability score of 0.720 ± 0.129, while *Serratia marcescens* was assigned with a score of 0.628 ± 0.108. Detailed bioinformatic protocols are available as Supplemental Information 1.

We chose several available bird microbiomes to compare against cockatiel, and all the sequences were downloaded from the declared repositories of their papers, and all of the sequences were processed with the same QC applied to cockatiel (Supplemental Information 1.). The compared samples are as follows: endangered psittacine bird samples, including three samples from the kakapo (*Strigops habroptilus)* fecal microbiome analyzed using the 454 V4–V5 region (Waite, Deines & Taylor, 2012); three turkey gut microbiome samples analyzed using the Illumina^®^ MiSeq V3 region (Danzeisen et al., 2015); three chicken (*Gallus gallus domesticus*) gut microbiome samples analyzed using the 454 V1–V3 regions (Stanley et al., 2013); one wild duck (*Aythya americana)* fecal microbiome sample analyzed using the IonTorrent V4 region (Strong et al., 2013); three samples from the emu (*Dromaius novaehollandiae)* cecal microbiome analyzed using 454 V3–V5 (Bennett et al., 2013); and finally, we used three swine (*Sus scrofa domesticus*; Yorkshire/Hampshire breed) gut microbiome samples as an outgroup for comparative purposes, which were analyzed using 454 V3 sequencing (Looft et al., 2012). For the comparative bird microbiome dataset, each compared species samples were processed individually. The comparative analysis was performed up to the OTU level, but the results were summarized at the Family level to compare the overall microbe diversity in other granivorous birds, and a mammalian outgroup was included as a reference. There are technological differences for each dataset, and we proceed cautiously by processing each data set individually. Clustering and alignment were performed on a sample/species basis, then phylum abundance values were calculated from the taxonomy assignments, transformed to relative frequency for each sample and plotted in a histogram to compare the summaries of diversity. This was an alternative procedure owing to the poor performance of *pick_closed_otus.py*, which reduced the overall number of OTUs in the cockatiel samples (Supplemental Information 1). However, the pick closed OTU analysis is available as Fig. S2. The clustering and analytical procedures are described in detail as Fig. S3.

### Data accessibility

The raw sequencing data are available on the NCBI under the project accession PRJNA320285, and the following Short Read Archive (SRA) accessions: SRR3473941, SRR3473942, and SRR3473943. OTU tables and their taxonomic assignations are available on figshare: https://dx.doi.org/10.6084/m9.figshare.3470555.

## Results

Gut microbiomes from three adult cockatiels were studied by sequence analysis of the 16S ribosomal gene; the specimens were healthy and lived in captivity. A total of 3,727,900 paired-end sequences were obtained with an average of 1,242,633 sequences per specimen. An average of 98,405 sequences per bird were the result of our quality controls, leaving just the top 7% of the sequences for further analysis. A total of 295,217 sequences were clustered into 19,280 operational taxonomic units (OTUs) at a 97% sequence identity cut-off (Table 1). The vast majority (99.6%) of observed OTUs were taxonomically assigned with the Greengenes database, which includes sequences from environmental samples.

**Table 1:** The basic statistics of the microbiome of cockatiels: number of reads, OTUs and diversity indexes. Diversity indexes were calculated with phyloseq (McMurdie & Holmes, 2013), Breakaway R package (Willis, Bunge & Whitman, 2015), and CatchAll (Bunge et al., 2012).

	**Cockatiel 1**	**Cockatiel 2**	**Cockatiel 3**	**Total**
**OTUs total diversity**				
Total sequences (raw paired-end)	1,256,170	1,255,086	1,216,644	3,727,900
Total sequences (merged, after QC)	90,520	97,161	107,536	295,217
Observed OTUs	6,957	7,566	7,154	19,280
Assigned phylotypes (Greengenes DB)	6,932	7,537	7,129	19,206
Assigned phylotypes (Closed OTUs DB)	109	206	115	309
Chao1	32,790 ± 1,260	36,659 ± 1,364	42,278 ± 1,822	
Shannon	3.2	3.47	3.27	
Simpson	0.779	0.841	0.845	
Breakaway estimate diversity	169,674	29,391	8,502	
CatchAll	152,809	101,814	160,648	
*CatchAll best parametric model*	*ThreeMixedExp*	*FourMixedExp*	*ThreeMixedExp*	
**OTUs diversity without singletons**				
Observed OTUs	879	1,000	991	2,870
Chao1	976 ± 23.45	1119 ± 25.42	1138 ± 28.23	
Shannon	2.46	2.76	2.65	
Simpson	0.74	0.81	0.82	
Breakaway estimate diversity	933	1,068	1,088	
CatchAll	1,093	1,251	1,276	
*CatchAll best parametric model*	*ThreeMixedExp*	*ThreeMixedExp*	*TwoMixedExp*	

**Table 2:** The assigned phylotypes and OTU abundance in the cockatiel microbiome.

Phyllum	Class	Order	Family	Genus	OTUs number	Frequency^a^ (%)
Firmicutes					16,287	90.5
	*Erysipelotrichi*	*Erysipelotrichales*	*Erysipelotrichaceae*		*8,072*	*57.4*
	*Clostridia*	*Clostridiales*	*Lachnospiraceae*	*Clostridium*	*759*	*17.1*
	*Bacilli*	*Lactobacillales*	*Lactobacillaceae*	*Lactobacillus*	*6,919*	*15.0*
	*Clostridia*	*Clostridiales*	*Clostridiaceae*	*Ca. Arthromitus*	*73*	*0.67*
Tenericutes					1,300	6.3
	*Mollicutes*	*Mycoplasmatales*	*Mycoplasmataceae*		*1,295*	*6.3*
Spirochaetes					754	1.5
	*Brevinematae*	*Brevinematales*	*Brevinemataceae*	*Brevinema*	*753*	*1.5*
Proteobacteria					732	1.4
	*γ* −*Proteobacteria*	*Enterobacteriales*	*Enterobacteriaceae*	*Serratia*	*359*	*1.0*
Cyanobacteria					41	0.191
Bacteroidetes					60	0.036
Actinobacteria					22	0.024
Other					83	0.051
**Total**					**19,279**	**100%**

**Notes.**

a A total of 295,217 sequencing reads were clustered into 19,280 non-redundant OTUs using a 97% identity threshold. The frequency column was calculated using the total number of sequencing reads assigned to a phylotype or OTU for each described level.

There are approximately 7,000 different OTUs for each cockatiel. The calculated Chao1 diversity index indicates an expected richness from 32,000 to 44,000 OTUs; this implies that many other OTUs can be found. The Simpson index implies that for all three cases there are few predominant bacteria, so OTUs not found in this study are in very low proportions (Table 1). Owing to the large difference between observed OTUs and Chao1, we did a standard microbiome procedure that involves removing the singletons from further analysis, and in this way, we found that the differences are not significant (*χ*^2^ = 0.32; *d*.*f*. = 2; *p* = 0.8521; Table 1). Most authors prefer to rarefy and use proportions to compare metagenomic data, which produces higher false positive rates (McMurdie & Holmes, 2014). Therefore, we include the diversity estimators without singletons, but we prefer to describe the overall found diversity, which includes its rare biosphere members; all further analyses were based on the complete OTU table (including singletons). Relative frequency analysis shows that the vast majority of organisms were *Firmicutes*, 57% of the sequences correspond to the *Erysipelotrichaceae* family, while 17 and 15% correspond to the *Clostridium* and *Lactobacillus* genus, respectively. *Proteobacteria* together stand for less than 1.5% of the observed bacteria, and *Tenericutes* represent 6.3%, while *Bacteroidetes* are less than 0.05%. The groups that showed greater diversity were *Erysipelotrichaceae* (8,072), *Lactobacillus* (6,919) and *Mycoplasmataceae* (1,295) (Table 2).

**Figure 1:**
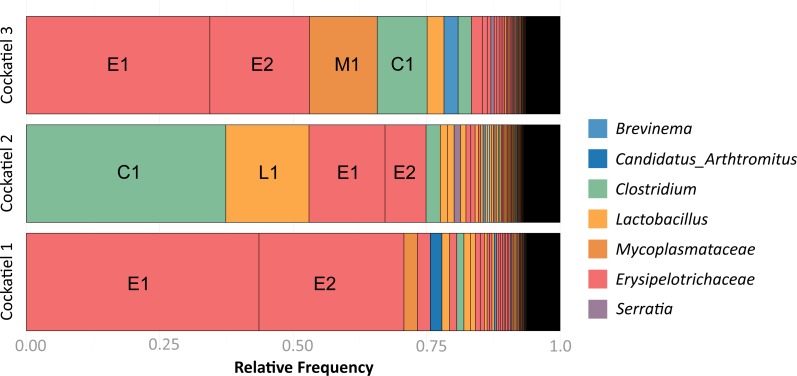
Frequency of OTUs for each cockatiel. The three cockatiels are clearly dominated by *Firmicutes Clostridium* OTU C1 overrepresentation is accompanied by a decrease of *Erysipelotrichaceae* E1 and E2 and an increase in *Lactobacillus* L1. Most OTUs are at very low frequency (<0.04). Cockatiel 1 is from “Local 2” breeder, cockatiels 2 and 3 are from “El refugio” breeder.

**Figure 2:**
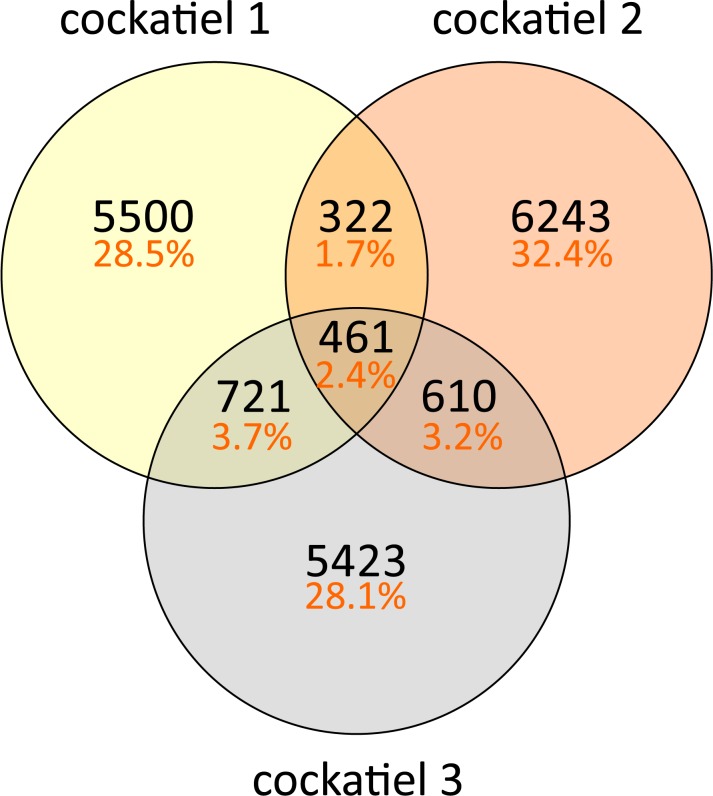
Shared and unique OTUs in cockatiel individuals. Most observed OTUs are unique for each cockatiel; however, shared OTUs are found at a higher frequency and correspond to more than 80% of the sequences. Cockatiel 1 is from “Local 2” breeder, and cockatiels 2 and 3 are from “El refugio” breeder.

The composition of bacteria per cockatiel is presented in a bar plot graph, which shows that there are dominant bacterial OTUs in each sample (Fig. 1); *Erysipelotrichaceae, Clostridium* and *Lactobacillus* are the most abundant groups. It is interesting to note that although more than 8,000 different *Erysipelotrichaceae* OTUs were found, only two OTUs were clearly dominant in the three samples; we named these E1 (OTU ID 3) and E2 (OTU ID 12). Likewise, the *Lactobacillus* OTU L1 (OTU ID 14) is the most common for all three birds, and the same occurs with *Clostridium colinum* OTU C1 (OTU ID 18651). We can also observe that 75% of the sample is made up of only 4 different bacteria in all cases. The Venn diagram shows that only 461 out of 19,279 OTUs are shared in all samples (Fig. 2). However, these shared bacteria are predominant, corresponding to 82, 83, and 85% of the microbiome of each bird. The 17,166 unique OTUs for each cockatiel are low frequency ones. For cockatiels 2 and 3 each exclusive OTU represents less than 0.1%; whereas Candidatus Division *Arthromitus* is exclusive to cockatiel 1, and its abundance is 1.9%.

We performed the standard QIIME *pick_closed_otus.py* script strategy to compare with other birds’ microbiome datasets, which uses a closed reference DB and discards any non-matching sequence for further analysis. When we tried to identify the sequences using the closed OTU database, which only includes type species, only 309 cockatiel OTUs were designated (Table 1); this indicates that most bacteria from the cockatiel gut microbiomes of the cockatiels are not found within the reference sequence models used by *pick_closed_otus.py*, but they are common in other environments (see Supplemental Information 1). According to previous work, closed OTU picking performs poorly if there is no sequence match in the reference database making for it impossible to identify new biodiversity (Rideout et al., 2014). This also indicates that *pick_closed_otus.py* is not the best way to analyze and compare microbiomes, although it is the recommended approach for comparing different microbiome studies using different 16S gene variable regions, sequencing coverage, and read lengths derived from independent experiments (Caporaso et al., 2010). To overcome the low number of *pick_closed_otus.py* assigned OTUs and perform the comparison, the raw datasets for the other bird microbiomes were downloaded and processed similarly to the cockatiels (see ‘Methods,’ and Supplemental Information 1).

## Discussion

**Figure 3:**
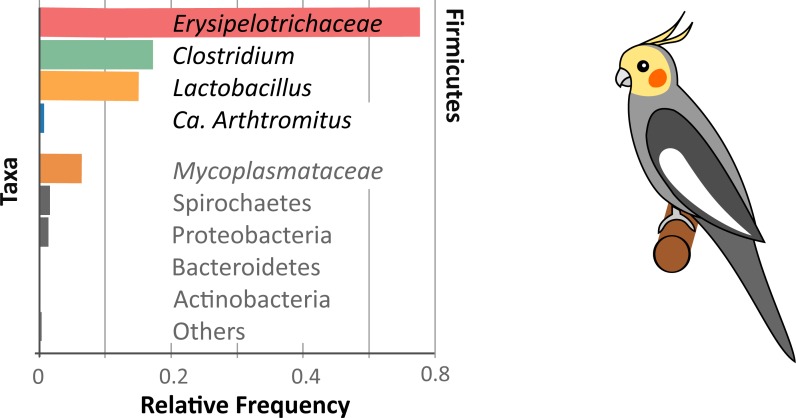
Cockatiel microbiome summary. *Firmicutes* dominate the microbiome diversity with *Erysipelotrichaceae, Clostridium, Lactobacillus*, and Ca. *Arthromitus*. On a lower scale is the *Tenericutes* phylum, with *Mycoplasmataceae* as the most abundant family. Other phyla such as *Spirochaetes, Proteobacteria, Bacteroidetes*, and *Actinobacteria* are barely detectable in this study.

A graphical summary of the microbiome diversity of the cockatiel is shown in Fig. 3, where the *Erysipelotrichaceae* family is highly dominant (57%) in the gut microbiomes of the cockatiels. This family is ubiquitous, and most known strains are avirulent. They are Gram positive bacteria, and there are both aerobic and anaerobic species. *Erysipelothrix rhusiopathiae* was first described in 1876 by Koch; however, many details of its physiology are unknown. The genus *Erysipelothrix* is aerobic; *E. rhusiopathiae* causes erysipelas disease in swine and poultry and also infects other animals including humans. Treatment with penicillin is sufficient to treat erysipelas, and in some countries it is common practice to vaccinate swine against the bacteria (Eamens, Forbes & Djordjevic, 2006). *E. tonsillarum*, unlike *E. rhusiopathiae*, can ferment sucrose and is not virulent, yet the 16S ribosomal gene diverges only at three bases (99.8% identical) (Kiuchi et al., 2000); the former prevents their differentiation in molecular studies such as this. Very little is known about *Erysipelotrichaceae* anaerobic species; they are found in the gut and oral microbiome of healthy humans and mice, and some species have been associated with periodontitis and halitosis (Verbarg et al., 2014). Cockatiel OTUs assigned as belonging to *Erysipelotrichaceae* did not have a reliable taxonomic assignment beyond the genus level, so we are unable to determine if they are aerobic or anaerobic or if they could be related to the known pathogenic species.

Lactobacilli are often in the gut microbiome of animals, and they can be used as probiotics to promote weight gain in chickens as well as to protect against some enteric bacteria, such as *Salmonella* or *Campylobacter* (Patterson & Burkholder, 2003). In this work, *Lactobacillaceae* represents 15% of the microbiome on average. The most common species (as best BLAST hits) for cockatiels are *Lactobacillus coleohominis, L. reuteri* and *L. acidipiscis*.

The three cockatiels from two different farms presented *Clostridium*, with *Clostridium colinum* as best BLAST hit assignment, asymptomatically with very different proportions: 1.3% (cockatiel 1; “Local 2” breeder), 37.1% (cockatiel 2; “El refugio” breeder), and 11.3% (cockatiel 3; “El refugio” breeder), respectively. *C. colinum* is a Gram positive anaerobic bacterium that has been identified as a pathogen in poultry; it causes ulcerative enteritis, also called quail disease, with symptoms including liver and spleen injury (Berkhoff, 1985). *C. colinum* infection can cause death within 2–3 days in bobwhite quail, while in other birds it can cause anorexia, humped backs, and watery or bloody diarrhea. Mortality in chickens is relatively low (2–10%), and birds usually recover in a couple of weeks. Ulcerative enteritis is associated with high population density and can be treated with streptomycin (Cooper, Bueschel & Songer, 2013). Our data indicate that *Clostridium colinum* is frequently found in the cockatiel gut microbiome, causing disease in birds only when out of control (Berkhoff, 1985; Kondo, Tottori & Soki, 1988; Perelman et al., 1991); this could also suggest that there are some low pathogenic OTUs, or they could increase their numbers with microbiome dysbiosis causing infections, or they are repressed by the immune system of the host.

The family *Mycoplasmataceae* constitutes 6.3% of the microbiome of cockatiels. In the Greengenes DB taxonomic classification, they are part of Phylum *Tenericutes*, but many other classifications consider them part of Phylum *Firmicutes*. *Mycoplasmas* are bacteria without cell walls that are usually located in the gut. *Mycoplasma* blooms are associated with diets rich in simple carbohydrates and thus are related to obesity in mice and humans, with these blooms displacing *Bacteroidetes* (Turnbaugh et al., 2006). The proportion observed in cockatiels is the same as in healthy chickens (*Gallus gallus*); for *Clostridium perfringens* infected chickens, the *Mycoplasma* proportion is increased 3.7 times (Stanley et al., 2013).

Of the bacteria in just one individual cockatiel (cockatiel 1, OTU ID 19091), we found “*Candidatus* division *Arthromitus*” with a frequency of almost 2%. Ca. *Arthromitus* is a segmented, filamentous non-culturable Gram positive bacterium; the filaments are anchored to the intestinal epithelium, and they are important for development of the mouse immune system (Talham et al., 1999). The fully sequenced Ca. *Arthromitus* genome shows a reduced genome, suggesting a close and lasting relationship with their host (Bolotin et al., 2014). In turkeys, they have been described as part of normal bacterial succession that becomes established around week 6; Ca. *Arthromitus* has also been linked to weight gain because they displace some types of *Lactobacilli* (Danzeisen et al., 2013). In the cockatiel, we do not observe clear displacement of *Lactobacilli*; however, as in the aforementioned paper, not all the birds show this bacterium.

The cockatiel most abundant *Proteobacteria* was *Serratia,* with a best BLAST hit for *S. marcescens*, which is usually located in water and food, but it is also a nosocomial pathogen that can cause respiratory and urinary infections, meningitis, endocarditis, etc. (Hejazi & Falkiner, 1997). Its frequency is not negligible, as it represents 1% of the observed bacteria in cockatiels.

We specifically looked for some potential pathogenic bacteria in the microbiomes of cockatiels. *Escherichia, Shigella, Mycobacterium, Chlamydia, Mycoplasma* and *Pasteurella* were not detected, while *Salmonella*, *Helicobacter, Campylobacter, Klebsiella, Staphylococcus, Aeromonas, Proteus, Listeria* and *Enterococcus* were found in just one animal to a lesser extent, at 1 ×10^−5^. *Pseudomonas* species that were found are not pathogenic to animals; we also detected *Streptococcus* in cockatiel 2 with a 0.1% frequency. *Streptococcus* is found at a lower frequency in cockatiels, and it was only detected in one bird.

**Figure 4:**
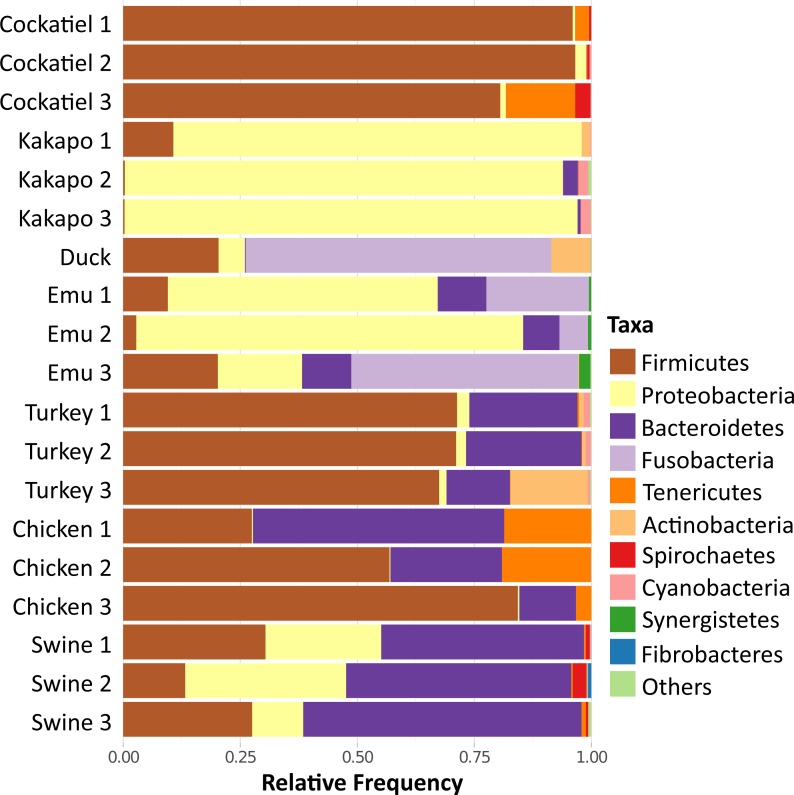
Relative frequency of bacteria in fecal microbiome of selected granivorous and omnivorous birds. The most common phyla in grain-eating birds are *Firmicutes, Proteobacteria* and *Bacteroidetes*. In cockatiels, only *Firmicutes* are dominant. *Tenericutes* are common in cockatiels and chickens, while *Fusobacteria* are in greater proportion in emu and duck. Selected poultry, and captive birds are mostly granivorous, in wild conditions they are feeding in vegetable matter and small animals (insects, snails, etc.).

Because of the importance of the type of diet for the development of the microbiome, the cockatiel fecal microbiomes were compared with those of other granivorous birds (kakapo, emu, duck, turkey, chicken) and with swine (Yorkshire/Hampshire breed) fecal microbiomes as a mammalian outgroup (Fig. 4). The comparison involves generating independent OTUs for each microbiome and then summarizing the results as phylum compositions. The most abundant phyla for cockatiels are as follows: *Firmicutes* (91%), followed by *Tenericutes* (5.9%), *Spirochaetes* (1.4%), and *Proteobacteria* (1.3%). *Firmicutes* are in high abundance (>50%) in turkeys and chickens, but within these farm birds the second most abundant phylum is *Bacteroidetes*, which is negligible in cockatiels. cockatiels and chickens have unusually higher average amounts of *Tenericutes* (cockatiels = 5.9%; chickens = 13%) compared with the rest of the analyzed bird species (average = 3%). *Bacteroidetes* are ubiquitous in bird microbiomes (average = 15.62%), but they are in low frequency in cockatiels (0.3%), kakapo (>1%), and in the wild duck (*Aythya americana* = 0.15%). The amount of *Bacteroidetes* is higher in turkey (average = 8.51%), chicken (average = 29.85%), and in the mammalian outgroup pig (average = 50.25%); these three cases are overcrowded and extremely sedentary farm animals. The cockatiel comparison with the kakapo is the most obvious as they are also parakeets. Kakapo are free-living endangered birds that are in a nation-wide conservation program, and the microbiome of the kakapo is composed mostly of *Proteobacteria* (79.61%) while in cockatiels, they are just 1.3%. Duck and emu have a high abundance of *Fusobacteria* (duck = 57%, emus = 24.67%), and this has also been observed in birds with a carnivorous diet, such as penguins and vultures (Dewar et al., 2013; Roggenbuck et al., 2014). When comparing the swine microbiome to those of the birds, we can observe that the swine are dominated by *Bacteroidetes* (50.25%), followed by *Proteobacteria* and *Firmicutes* in equivalent amounts, both having an average of ∼23%.

**Figure 5:**
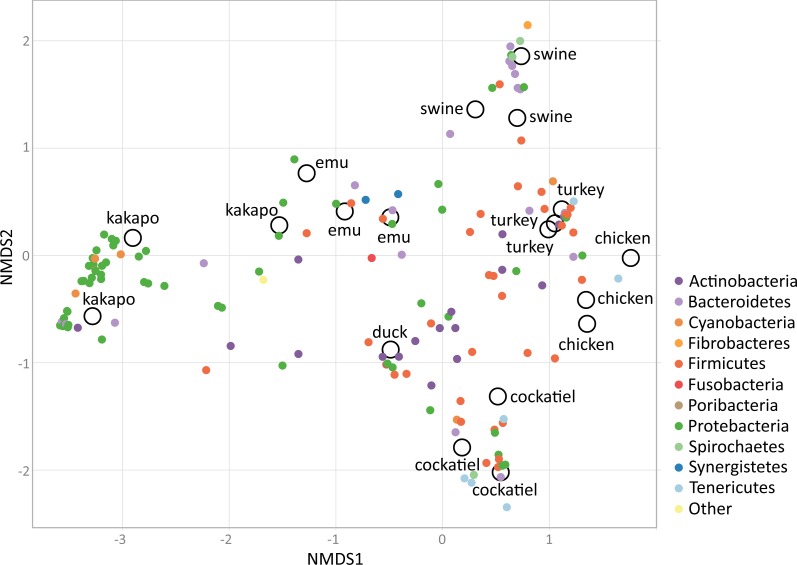
Non-metric dimensional scaling (NMDS), using Bray–Curtis dissimilarities, biplot analysis for the bird microbiomes. There is no cluster attraction due to the sequencing technology used or 16S rRNA gene region sequenced. Swine microbiomes are used for outgroup purposes only, and they are clustered apart owing to their *Bacteroidetes*. Interestingly, the ordination has most *Proteobacteria* on the left, and *Firmicutes* on the right quadrant. There are clusters of poultry-related samples of different species, such as the case for turkey, chicken, and emu. Kakapo are clustered separately from every other species on the left quadrant and are clearly being clustered by its *Proteobacteria* abundance. The wild duck also clusters separately owing to its particular microbiome configuration with *Actinobacteria, Fusobacteria*, and *Proteobacteria*. Finally, cockatiels are in the bottom right quadrant, being clustered separately owing to their *Firmicutes, Tenericutes, Spirochaetes*, and some *Proteobacteria* families. The stress level for NMDS = 0.1429879. This is a coarse comparison at phyla level between microbiome samples of different species, fine detail could be obtained only through comparing samples using the same sequencing technology, and coverage.

Using non-metric dimensional scaling (NMDS), the bird samples were clustered according to their microbiome taxonomic profiles (Fig. 5). The clustering was performed using family level taxa summaries (*N* = 203), with each individual dataset being taxonomically assigned independently. The clustering analysis includes different original studies that used diverse methodologies. The most important methodological considerations are sequencing technology and the 16S rRNA gene region used in each study: the cockatiel was analyzed using V3–V4; emu, turkey, and pig used V3; kakapos and wild duck used V4. Most of the samples were sequenced by 454 pyrosequencing, but the wild duck was IonTorrent, and MiSeq was the technology for the turkey and cockatiel sequences. From the NMDS cluster patterns as well as several clustering methods (CCA, DCA, MDS, NMDS, PCoA, and RDA; available as Fig. S1), it seems that there is no cluster attraction because of the sequencing technology or because of the 16S rRNA gene region used. The poultry bird species and the wild duck (*Aythya americana*) microbiomes cluster closer to the center and are dominated by *Firmicutes, Actinobacteria,* and *Tenericutes* for chickens and turkeys; the emu microbiome hosts a middle ground between *Firmicutes* and *Proteobacteria* in the center of the clustering; the Kakapo microbiomes cluster separately from every other bird and are dominated by *Proteobacteria* species; the wild duck microbiomes are mainly *Actinobacteria, Firmicutes,* and *Proteobacteria;* the cockatiel microbiomes cluster to the lower right quadrant and are dominated by *Firmicutes, Tenericutes, Spirochaetes,* and some *Proteobacteria.* Swine are used as a comparative outgroup, and their microbiomes host large amounts of *Bacteroidetes.*

It appears that *Bacteroidetes* species are not major players in at least three bird samples analyzed here: cockatiels, kakapos, and wild ducks. *Bacteroidetes* species could be an addition to the bird microbiome due to poultry management, but they appear to be reduced in wild birds and parrots, which are not being selected for rapid weight gain. In mammals such as the mouse, the ∼50% increase in *Firmicutes* and the corresponding decrease in *Bacteroidetes* abundance is connected to an obese mouse phenotype. The rise of *Firmicutes* in obese mice is connected to an increased ability to harvest energy from their diet (Turnbaugh et al., 2006), and it is also correlated with geographical variation (latitude) (Suzuki & Worobey, 2014). The *Firmicutes* richness in the microbiomes of wild birds and cockatiels could also be connected to more efficient energy harvesting capabilities, as the increase in *Bacteroidetes* frequency in the poultry birds could be directed by extensive human manipulation. However, additional work is needed to further compare phylum abundance among a wider set of cockatiels as well as raised poultry, and wild living birds.

## Conclusions

The fecal microbiomes of cockatiels are the initial descriptors of bacterial diversity in this pet bird that is distributed across all continents. We were able to estimate a total of 19,280 unique OTUs in all of the sampled cockatiels. The OTU diversity is not evenly distributed, as the cockatiel microbiome is clearly dominated by *Firmicutes*, especially the *Erysipelotrichaceae* family. Some probable pathogenic bacteria, such as *Clostridium* sp. (probably *C. colinum*) with an average abundance in the samples of 17.13% and *Serratia* sp. (probably *S. marcescens*) with an average of 1.04%, were found in every cockatiel sample, suggesting that they are frequent inhabitants in the fecal microbiota of healthy cockatiels, whereas other pathogenic bacteria are not found regularly in cockatiels. Conversely, other possible pathogenic bacteria such as *Escherichia, Shigella, Mycobacterium, Chlamydia, Mycoplasma* and *Pasteurella* were not found in any of the cockatiel samples. Finally, when comparing the fecal microbiomes of cockatiels to those of other granivorous birds, it becomes evident that *Firmicutes* dominates the cockatiel fecal microbiome, with a very low *Proteobacteria* abundance that is similar to that found in chicken and turkey fecal microbiomes. Cockatiels have quite reduced numbers of some widespread phyla found in several birds (e.g., *Bacteroidetes*).

##  Supplemental Information

10.7717/peerj.2837/supp-1 Supplemental Information 1 Cockatiel microbiome bioinformatic protocols

10.7717/peerj.2837/supp-2 Table S1 CatchAll and breakaway diversity estimations

10.7717/peerj.2837/supp-3 Figure S1 Multiple ordinations on avian microbiomes

10.7717/peerj.2837/supp-4 Figure S2 Closed otus phyla barplot

10.7717/peerj.2837/supp-5 Figure S3 Closed otus phyla NMDS ordination

## Additional Information and Declarations

### DNA Deposition

The following information was supplied regarding the deposition of DNA sequences: Raw sequencing data is available on the NCBI under the project accession PRJNA320285, and the following Short Read Archive (SRA) accessions: SRR3473941, SRR3473942, and SRR3473943.

### Data Availability

The following information was supplied regarding data availability: Alcaraz, Luis David; Hernàndez, Apolinar M; Peimbert, Mariana (2016): Fileset: Exploring the cockatiel (*Nymphicus hollandicus*) fecal microbiome, bacterial inhabitants of a worldwide pet. figshare. https://dx.doi.org/10.6084/m9.figshare.3470555.v1.
