# Supplementary material for: Exploring the cockatiel (Nymphicus hollandicus) fecal microbiome, bacterial inhabitants of a worldwide pet
Source: PeerJ. 2016 Dec 22;4:e2837. doi: 10.7717/peerj.2837 (PMC5183021; doi:10.7717/peerj.2837)
Supplement: Supplemental Information 1 [file peerj-04-2837-s001.docx]

Cockatiel microbiome bioinformatic protocols.

**Author names and affiliations**

Luis David Alcaraz^a^, Apolinar M. Hernández^b^, and Mariana Peimbert^b,^
^a^ Laboratorio Nacional de Ciencias de la Sostenibilidad. Instituto de Ecología. Universidad Nacional Autónoma de México. AP 70-275, Ciudad Universitaria, UNAM, 04510, Cd. Mx., México.

^b^ Departamento de Ciencias Naturales. Universidad Autónoma Metropolitana, Unidad Cuajimalpa. Av. Vasco de Quiroga 4871, Col. Santa Fe Cuajimalpa, 05348, Cd. Mx., México.

1. Preprocessing, assembly, QC, OTU clustering, taxonomy assignments.

Raw MISeq sequences were assembled with Pandaseq and Quality Control with fastx_tools generating a Sun Grid Engine job to be submitted to our deep-thought cluster, by the following script :

| #!/bin/bash  SEQS=/home/run_ago_15 SALIDAS=/home/salidas BIN=/home/bin BIN2=/home/bin COUNT=0 for FAA in `cat lista` do let COUNT=COUNT+1 echo "#!/bin/bash" >$*.$COUNT.scr echo "#$ -cwd" >>$*.$COUNT.scr echo "#$ -j y" >>$*.$COUNT.scr echo "#$ -S /bin/bash" >>$*.$COUNT.scr echo "zcat $SEQS/$FAA"_R1.fastq.gz" \| $BIN2/fastx_trimmer -l 250 > $SEQS/$FAA"tr_R1.fastq" &" >>$*.$COUNT.scr  echo "zcat $SEQS/$FAA"_R2.fastq.gz" \| $BIN2/fastx_trimmer -l 250 > $SEQS/$FAA"tr_R2.fastq"" >>$*.$COUNT.scr echo "$BIN/pandaseq -B -f $SEQS/$FAA"tr_R1.fastq" -r $SEQS/$FAA"tr_R2.fastq" -t 0.95 -l 250 -L 470 -o 15 -w $SALIDAS/$FAA"_4nov15.fasta" -G $SALIDAS/$FAA"-4nov15.log.bz2"" >>$*.$COUNT.scr  done |
| --- |

The manual of pandaseq is available here: <http://neufeldserver.uwaterloo.ca/~apmasell/pandaseq_man1.html>

Particularly the following options were modified:

−o minoverlap

Sets the minimum overlap between forward and reverse reads. By default, this is at least one nucleotide of overlap. Raising this number does not generally increase the quality of the output as alignments with small overlaps tend to score poorly and are discarded anyway. This was chosen to be a value of 15, rather than 1.

−O maxoverlap

Sets the maximum overlap between forward and reverse reads. By default, this is the read length. In highly overlapping sequences (i.e., those where the end of one read precede the start of the other), this parameter should be set to the sum of the input reads, or a value larger than that. The maxoverlap was set to default which is the read length

−t threshold

The score, between zero and one, that a sequence must meet to be kept in the output. Any alignments lower than this will be discarded as low quality. Increasing this number will not necessarily prevent uncalled bases (Ns) from appearing in the final sequence. It is also used as the threshold to match primers, if primers are supplied. The default value is 0.6. Our chosen value was 0.95 in a 0-1 scale.

Length was filtered up to a size of 470 bp because the expected amplicon size is around 460 bp from the 16S rRNA gene V3-V4 primers (<http://support.illumina.com/content/dam/illumina-support/documents/documentation/chemistry_documentation/16s/16s-metagenomic-library-prep-guide-15044223-b.pdf>).

Afterwards, quality of the assembly was visualized by FastQC and their html generated reports.

Clustering of the OTUs at the 97% identity was done with cd-hit-est

| $bin/cd-hit/cd-hit-est -c 0.97 -T 0 -i ~/names.fas -o ~/output.clstr |
| --- |

Then the cluster file is converted to a file that looks exactly the same than the output of QIIME’s (v1.9.0) pick_otus:

| $perl -pne 's/\t//g;s/^.*,//g;s/\.\.\..*$//g;s/\n/\t/g;s/\>Cluster\ /\n/g;s/\>//g; eof && do{chomp; print "$_ \n"; exit}' output.clstr >cockatiel.otu  $sed -i '1d' cockatiel.otu |
| --- |

With the previous OTU file use QIIME’s pick_rep_set.py to select representative *de novo* picked OTUs:

| $pick_rep_set.py -i cockatiel.otu -f seqs.fna -o rep_set1.fna |
| --- |

Parallel assigning taxonomy:

| $parallel_assign_taxonomy_blast.py -i rep_set1.fna -o taxonomy -r /qiime/gg_otus-13_8-release/rep_set/97_otus.fasta -t //qiime/qiime_software/gg_otus-13_8-release/taxonomy/97_otu_taxonomy.txt |
| --- |

BlastTaxonAssigner parameters:

Application:blastn/megablast

Max E value:0.001

Min percent identity:90.0

blast_db:/home/salidas/perico_tax/BTA_L2SRA_/97_otus.fasta

id_to_taxonomy_filepath:/home/qiime/1.9.0/software/gg_otus-13_8-release/taxonomy/97_otu_taxonomy.txt

Number of sequences inspected: 97

Number with no blast hits: 0

Result path: /tmp/3820.1.lda.q/assign-taxMcWg8H

BlastTaxonAssigner parameters:

Application:blastn/megablast

Max E value:0.001

Min percent identity:90.0

Build OTU table:

| $make_otu_table.py -i cockatiel.otu -t perico_tax/perico_repset_tax_assignments.txt -o perico.biom |
| --- |

Then the otu table is converted to text along with the taxonomy assignments:

| $biom convert --to-tsv -i perico.biom -o ave.txt --table-type "Taxon table" --header-key=taxonomy |
| --- |

To split the taxonomy from the OTU table:

| $perl -pe 's/\; /\;/g' ave.txt \| awk '{print $1,"\t",$NF}' \| perl -pe 's/\;/\t/g' >aves.tax |
| --- |

To clean up the OTU table in txt (tab separated) format:

| $rev ave.txt \| cut -f2- \| rev >ave.biom.txt |
| --- |

To prepare OTU table header to be loaded in phyloseq:

| $cat ave.biom.txt \| grep -v "biom file" \| perl -pe 's/\#OTU\ ID/OTU_ID/g' >ave.biom.clean.txt |
| --- |

Cleaning up the OTU table. First step, remove OTUs with no matches to the 16S rRNA gene database (Greengenes):

| $grep "no_hit" perico.tax.txt \| cut -f1 \| while read x; do echo "sed -i.bak '/$x/d'" perico.clean.biom.txt; done \| bash  $grep -v "no_hit" perico.tax.txt >perico.clean.tax #to clean no hits from the taxonomy file |
| --- |

Cleaning up the OTU table. Second step, remove Chimeras, identified by ChimeraSlayer:

| $parallel_align_seqs_pynast.py -i ~/ninfa/ninfa.repset.fna -o ~/ninfa/ninfa.align -X ninfa  $parallel_identify_chimeric_seqs.py -i ~/ninfa/ninfa.repset_aligned.fasta -r ~/qiime_default_reference/gg_13_8_otus/rep_set_aligned/85_otus.fasta -o ~/ninfa/ninfa.chimera.txt  #generate a list of OTUs to be removed  $cat ninfa.chimerablast.txt \| awk '{print $1}' >remove  #Remove Chimeric OTUs  $for N in `cat remove`; do egrep -v \"$N \" test >test.t; mv test.t test; done |
| --- |

Cleaning up the OTU table. Third step, remove known contaminants (mitochondria, chloroplasts)

| $grep -i "chloroplast" perico.tax.txt \| cut -f1 \| while read x; do echo "sed -i.bak '/$x/d'" perico.clean.biom.txt; done \| bash $grep -iv "chloroplast" perico.tax.txt >perico.clean.tax  $grep -i "mitochondria" perico.tax.txt \| cut -f1 \| while read x; do echo "sed -i.bak '/$x/d'" perico.clean.biom.txt; done \| bash $grep -iv "mitochondria" perico.tax.txt >perico.clean.tax |
| --- |

**The metadata file order does matter, check the OTU table sample order and the metadata should be exactly in the same order for phyloseq to load them correctly.

2. R analysis

All statistical analysis were done in R. R version 3.2.1 (2015-06-18; x86_64). With the following packages and versions:

[1] Rcpp_0.12.0 locfit_1.5-9.1 ape_3.3

[4] Biostrings_2.36.1 digest_0.6.8 foreach_1.4.2

[7] biom_0.3.12 GenomeInfoDb_1.4.1 plyr_1.8.3

[10] chron_2.3-47 futile.options_1.0.0 acepack_1.3-3.3

[13] stats4_3.2.1 RSQLite_1.0.0 ggplot2_1.0.1

[16] zlibbioc_1.14.0 data.table_1.9.4 annotate_1.46.1

[19] vegan_2.3-0 S4Vectors_0.6.3 rpart_4.1-10

[22] Matrix_1.2-4 proto_0.3-10 splines_3.2.1

[25] BiocParallel_1.2.9 geneplotter_1.46.0 stringr_1.0.0

[28] foreign_0.8-66 igraph_1.0.1 munsell_0.4.2

[31] BiocGenerics_0.14.0 multtest_2.24.0 mgcv_1.8-12

[34] nnet_7.3-12 gridExtra_2.0.0 Hmisc_3.16-0

[37] IRanges_2.2.5 codetools_0.2-14 XML_3.98-1.4

[40] MASS_7.3-45 grid_3.2.1 nlme_3.1-126

[43] xtable_1.7-4 gtable_0.1.2 DBI_0.3.1

[46] magrittr_1.5 scales_0.2.5 stringi_0.5-5

[49] XVector_0.8.0 reshape2_1.4.1 genefilter_1.50.0

[52] RcppArmadillo_0.5.300.4 latticeExtra_0.6-26 futile.logger_1.4.1

[55] Formula_1.2-1 lambda.r_1.1.7 iterators_1.0.7

[58] tools_3.2.1 RJSONIO_1.3-0 ade4_1.7-2

[61] Biobase_2.28.0 DESeq2_1.8.1 parallel_3.2.1

[64] survival_2.38-3 AnnotationDbi_1.30.1 colorspace_1.2-6

[67] cluster_2.0.3 GenomicRanges_1.20.5

The packages must be installed and loaded to reproduce the results.

| #Data loading  library(phyloseq)  library(ggplot2)  otu <- as.matrix(read.table("perico.clean.biom.txt", header=T, row.names=1))  OTU = otu_table(otu, taxa_are_rows=T)  taximat = as.matrix(read.table("perico.tax.txt", header=T, row.names=1))  taxi=tax_table(taximat)  ninfa = phyloseq (OTU, taxi)  Ninfa_data =read.table(“data.txt”, header=T, row.names=1))  sampledata = sample_data(data.frame(id=ninfa_data$id, sampling=ninfa_data$sampling, row.names=sample_names(ninfa)))  ninfa = phyloseq (OTU, taxi, sampledata)  ninfaFilter = ninfa  ninfa0 = genefilter_sample(ninfaFilter, filterfun_sample(function(x) x > 5), A =  0.5 * nsamples(ninfaFilter))  ninfa1F = prune_taxa(ninfa0, ninfaFilter)  p1 <- plot_bar(ninfa1F, fill="Phylum", facet_grid=~Genus)  ggsave ("genus_bar.pdf", p1)  library(vegan)  data(BCI)  otuninfa2 <- t(read.table("perico.clean.biom.txt", header=T, row.names=1))  rarecurve(otuninfa2, step=10000)  col <- c("blue", "orange", "green")  lty <- c("solid", "dashed", "dotdash")  pars <- expand.grid(col = col, lty = lty, stringsAsFactors = FALSE)  out <- with(pars[1:3, ], rarecurve(otuninfa2, step=10, col = col, lty=lty))  pdf("rarefaction_onlyninfa.pdf")  p2 <- plot_bar(ninfa1F, fill="Genus", facet_grid=~Order) + theme_bw()  p2  ggsave("plot_barOrder_genus.pdf", p2)  #Venn diagrams / heatmaps  install.package("venn")  install.packages("venn")  library(venn)  library(limma)  vennDiagram(ninfa)  ninfa  otuninfa  vennDiagram(otuninfa)  pdf("vennNinfa.pdf")  top20OTUs <- names(sort(taxa_sums(ninfa), TRUE)[1:20])  ninfa20 <- prune_taxa(top20OTUs, ninfa)  ninfa20  otu_table(ninfa20)  ninfa20  tax_table(ninfa20)  plot_bar(ninfa20)  plot_bar(ninfa20, fill="Genus")  plot_heatmap(ninfa1F, "MDS", "bray", )  plot_heatmap(ninfa1F, "MDS", "jaccard", )  plot_heatmap(ninfa1F, "NMDS", "jaccard", )  plot_heatmap(ninfa1F, "PCoA", "jaccard", )  plot_heatmap(ninfa1F, "NMDS", "jaccard", )  plot_heatmap(ninfa1F, "NMDS", "jaccard", )  plot_heatmap(ninfa1F, "PCoA", "jaccard", )  plot_heatmap(ninfa1F, "CCA", "jaccard", )  plot_heatmap(ninfa1F, "RDA", "jaccard", )  plot_heatmap(ninfa1F, "DPCoA", "jaccard", )  plot_heatmap(ninfa1F, "CCA", "jaccard", )  plot_heatmap(ninfa1F, "CCA", "manhattan", )  plot_heatmap(ninfa1F, "CCA", "euclidean", )  plot_heatmap(ninfa1F, "NMDS", "euclidean", )  plot_heatmap(ninfa1F, "NMDS", "manhattan", )  plot_heatmap(ninfa1F, "CCA", "manhattan", )  plot_heatmap(ninfa1F, "NMDS", "manhattan", )  plot_heatmap(ninfa50, "NMDS", "manhattan", )  plot_heatmap(ninfa50, "CCA", "manhattan", )  plot_heatmap(ninfa50, "NMDS", "manhattan", )  plot_heatmap(ninfa20, "NMDS", "manhattan", )  plot_heatmap(ninfa50, "NMDS", "manhattan", )  plot_heatmap(ninfa100, "NMDS", "manhattan", )  m= plot_heatmap(ninfa100, "NMDS", "manhattan", )  ggsave ("ninfas_heatmap100.pdf" , m)  m= plot_heatmap(ninfa1F, "NMDS", "manhattan", )  ggsave ("ninfas_heatmap1F.pdf" , m)  ninfa.scale = transform_sample_counts(ninfa, function(x) x/sum(x))  ninfa.scale = prune_taxa(taxa_sums(ninfa.scale)>0, ninfa.scale)  #phylum barplots from summary taxa data  library(ggplot2)  library(phyloseq)  phylum <- read.table("otu_table_L2.txt", header=T, row.names=1)  abund_table <- t(phylum)  x <- abund_table[,order(colSums(abund_table), decreasing=T)]  for (i in 1:dim(x)[2]){  tmp<-data.frame(row.names=NULL,Sample=rownames(x),Taxa=rep(colnames(x)[i],dim(x)[1]),Value=x[,i])  if(i==1){df<-tmp} else {df<-rbind(df,tmp)}  }  dat$V1 <- factor(df$Sample, levels=(unique(rownames(x))))  N <- length(df$Taxa)  colours <- c("darkorange1", "snow3", "green3", "navy", "greenyellow", "purple", "turquoise4", "skyblue", "violetred", "yellow", "red", "purple4", "turquoise1", "violet", "slateblue4", "olivedrab", "tomato4", "aquamarine")  p <- ggplot (df, aes(Sample, Value, fill=Taxa)) + geom_bar(stat="identity")  p2 <- p + scale_fill_manual(values=colours[1:(N+1)])  p2 <- p2 +theme_bw() +theme(axis.text.x=element_text(angle=90, hjust=1, vjust=0.5))  p2 <- p2 + xlab("") + ylab("Relative Frequency")  ggsave("bar_phylum.pdf")  #ordination  library(phyloseq)  library(ggplot2)  aves <- as.matrix(read.table("phyloseq.txt", header=T, row.names=1))  OTU = otu_table(otu, taxa_are_rows=T)  taximat <- as.matrix(read.table("phyloseq.tax.txt", header=T, row.names=1))  taxi = tax_table(taximat)  aves = phyloseq(OTU, taxi)  aves_data=read.table("phyloseq.map.txt", header=T, row.names=1)  sampledata = sample_data(data.frame(id=aves_data$id, species=aves_data$species, row.names=sample_names(aves)))  gp.ord <- ordinate(aves, "NMDS", "bray")  p1 = plot_ordination(aves, gp.ord, type = "split", color = "Phylum", shape="species")  p1  p2 <- p1 + scale_shape_manual(values=c(16:25))  p2  p2 <- p1 + scale_shape_manual(values=c(16:25)) + theme_bw()  p2  p2 <- p1 + scale_shape_manual(values=c(16:25)) + theme_bw() + label="species"  p2 <- p1 + scale_shape_manual(values=c(16:25)) + theme_bw() + label="specie"  p1 = plot_ordination(aves, gp.ord, type = "split", color = "Phylum", shape="species", label = "species")  p1  p1 = plot_ordination(aves, gp.ord, type = "split", color = "Phylum", shape="species", label = "id")  p1  p2 <- p1 + scale_shape_manual(values=c(16:25)) + theme_bw()  p2  gp.ord2 <- ordinate(aves, "PCoA", "bray")  gp.ord3 <- ordinate(aves, "PCoA", "manhattan")  p2 = plot_ordination(aves, gp.ord2, type = "split", color = "Phylum", shape="species", label = "id") + scale_shape_manual(values=c(16:25)) + theme_bw()  p2  p3 = plot_ordination(aves, gp.ord3, type = "split", color = "Phylum", shape="species", label = "id") + scale_shape_manual(values=c(16:25)) + theme_bw()  p3  p1 = plot_ordination(aves, gp.ord, type = "split", color = "Phylum", shape="species", label = "id") + scale_shape_manual(values=c(16:25)) + theme_bw()  p1  p1 + geom_point(size=2)  p1 + geom_point(size=3)  ggsave("ordination_NMDS_bray.pdf", p1 + geom_point(size=3) + alpha = 0.6)  ggsave("ordination_NMDS_bray.pdf", p1 + geom_point(size=3+ alpha = 0.6))  ggsave("ordination_NMDS_bray.pdf", p1 + geom_point(size=3, alpha = 0.6))  library(plyr)  library(breakaway)  peri <- read.table("perico.clean.biom.txt", header=T, sep="\t", row.names=1)  breakaway((count(peri$S21)[-1,]))  breakaway((count(peri$S22)[-1,]))  breakaway((count(peri$S23)[-1,])) |
| --- |

3. Comparative datasets

The comparative set species were downloaded from the following SRA Accessions/URLs.

**Kakapo:**

[Kakapo captivity study-Adult7_2012](http://www.ncbi.nlm.nih.gov/sra/SRX385262%5Baccn%5D)

1 LS454 (454 GS FLX) run: 3,015 spots, 1.1M bases, 513,797b downloads

Accession:SRX385262

Select item 552349[ ] 2.

[Kakapo captivity study-Adult6_2012](http://www.ncbi.nlm.nih.gov/sra/SRX385261%5Baccn%5D)

1 LS454 (454 GS FLX) run: 1,958 spots, 743,227 bases, 351,204b downloads

Accession: SRX385261

Select item 552348[ ] 3.

[Kakapo captivity study-Adult5_2012](http://www.ncbi.nlm.nih.gov/sra/SRX385260%5Baccn%5D)

1 LS454 (454 GS FLX) run: 1,918 spots, 724,992 bases, 355,746b downloads

Accession: SRX385260

Select item 552347[ ] 4.

[Kakapo captivity study-Adult4_2012](http://www.ncbi.nlm.nih.gov/sra/SRX385259%5Baccn%5D)

1 LS454 (454 GS FLX) run: 2,720 spots, 1M bases, 482,105b downloads

Accession: SRX385259

**Turkey:**

Data was downloaded form the following link: <http://conservancy.umn.edu/handle/11299/174930>

Reference: <http://www.ncbi.nlm.nih.gov/pmc/articles/PMC4672264/>

**Chicken:**

Data was downloaded from the following project:

<http://metagenomics.anl.gov/metagenomics.cgi?page=MetagenomeProject&project=6177>

And from the following samples:

<http://metagenomics.anl.gov/metagenomics.cgi?page=DownloadMetagenome&metagenome=4537568.3>

<http://metagenomics.anl.gov/metagenomics.cgi?page=DownloadMetagenome&metagenome=4537569.3>

<http://metagenomics.anl.gov/metagenomics.cgi?page=DownloadMetagenome&metagenome=4537583.3>

**Wild duck (Aythya americana):**

Reference:<http://f1000research.com/articles/2-224/v2#article-reports>

Data are available as supplementary material to the paper: <https://ndownloader.figshare.com/files/1231298>

**Emu**

Reference: <https://www.researchgate.net/profile/Darin_Bennett/publication/249318695_Characterization_of_cecal_microbiota_of_the_emu_%28Dromaius_novaehollandiae%29/links/02e7e520ffa4f5fc10000000.pdf>

Downloaded from: <http://trace.ncbi.nlm.nih.gov/Traces/sra/sra.cgi?exp=SRX254725&cmd=search&m=downloads&s=seq>

**Pigs (outgroup):**

Reference: <http://www.ncbi.nlm.nih.gov/pmc/articles/PMC3277147/>

SRA project: <http://www.ncbi.nlm.nih.gov/sra/SRX038401>

Specific samples:

| 1 | [SRS151804](http://www.ncbi.nlm.nih.gov/biosample?term=SRS151804) : [pig 26-0](http://trace.ncbi.nlm.nih.gov/Traces/sra/sra.cgi?cmd=search&amp;m=search&amp;s=seq&amp;exp=SRX038401&amp;member=pig%2026-0) | 8,411 | 2.3M |
| --- | --- | --- | --- |
| 2 | [SRS151805](http://www.ncbi.nlm.nih.gov/biosample?term=SRS151805) : [pig 35-0](http://trace.ncbi.nlm.nih.gov/Traces/sra/sra.cgi?cmd=search&amp;m=search&amp;s=seq&amp;exp=SRX038401&amp;member=pig%2035-0) | 11,532 | 3.1M |
| 3 | [SRS151806](http://www.ncbi.nlm.nih.gov/biosample?term=SRS151806) : [pig 37-0](http://trace.ncbi.nlm.nih.gov/Traces/sra/sra.cgi?cmd=search&amp;m=search&amp;s=seq&amp;exp=SRX038401&amp;member=pig%2037-0) | 9,860 | 2.7M |

For each sequence of the comparative dataset each study was processed individually by the following commands:

| for N in *.fastq; do fastq_quality_filter -Q 33 -p 97 -q 20 -i $N \| fastx_trimmer -m 100 \| fastq_to_fasta -o $N.filter.fasta; done |
| --- |

Each file was renamed in a sequential header with a number for each sequence, in a way similar of QIIME’s output of split libraries with the following perl script:

| #!/usr/bin/perl  # este script cambia cada uno de los identificadores de un archivo fasta a >prefix_# donde prefix es el  # primer argumento del script y # es una numeración de cada una de las secuencias que componen el archivo el segundo argumento es el nombre del archivo fasta a renombrar.  # este script es útil si se quiere usar QIIME en archivos fasta desmultiplexados o individuales.  # uso: ./header.fasta.numbers.pl prefix nombre_del_archivo.fasta  # Luis David Alcaraz 2013-04-11  my $prefix = $ARGV[0]; chomp $prefix;  my $f = 1;  my $fasta_file = $ARGV [1]; chomp $fasta_file;  my $fh;  open($fh, $fasta_file) or die "can't open $fasta_file: $!\n";  open(OUT, ">$fasta_file.numbered.fas") \|\| die "can't open $fasta_file.numbered.fas\n";  my %sequence_data;  while (read_fasta_sequence($fh, \%sequence_data)) {  print OUT ">$sequence_data{header}\n$sequence_data{seq}\n";  }  close $fh;  close OUT;  sub read_fasta_sequence {  my ($fh, $seq_info) = @_;  $seq_info->{seq} = undef; # clear out previous sequence  # put the header into place  $seq_info->{header} = $seq_info->{next_header} if $seq_info->{next_header};  my $file_not_empty = 0;  while (<$fh>) {  $file_not_empty = 1;  next if /^\s*$/; # skip blank lines  chomp;  if (/^>/) { # fasta header line  my $h = $_;  $h =~ s/>/$prefix\_$f\ /;  $f++;  if ($seq_info->{header}) {  $seq_info->{next_header} = $h;  return $seq_info;    }  else { # first time through only  $seq_info->{header} = $h;  }  }  else {  s/\s+//; # remove any white space  s/\n\n/\n/;  $seq_info->{seq} .= $_;  }  }  if ($file_not_empty) {  return $seq_info;  }  else {  # clean everything up  $seq_info->{header} = $seq_info->{seq} = $seq_info->{next_header} = undef;  return;  }  } |
| --- |

Each species was concatenated independently (3 individuals per sample, all but wild duck).

Example:

| cat chicken/chickens.fna kakapo/kakapo.fna ninfa/perico.nov15.fasta turkey/turkey.fas wild_duck/duck.fna >comp-mic_aves.fna |
| --- |

There is need to generate a parameter_pick for pick_closed_reference.otus.py with the following information to enable reverse strand matching:

| $echo “pick_otus:enable_rev_strand_match True” -p parameter_pick |
| --- |

We made a regular QIIME’s pick_closed_reference.otus.py assignment, with the previous files :

| $pick_closed_reference_otus.py -f -i ~/comp-mic_aves.fna -p ~/parameter_pick -a -o ~/salidas/mic_aves/ |
| --- |

Pick closed otu parameters employed are described below:

-r REFERENCE_FP, --reference_fp=REFERENCE_FP

The reference sequences [default:

/home/qiime/1.9.0/lib/python2.7/site-package

s/qiime_default_reference/gg_13_8_otus/rep_set/97_otus

.fasta]. NOTE: If you do not pass -r to this script,

you will be using QIIME's default reference sequences.

In this case, QIIME will copy the corresponding

reference tree to the output directory. This is the

tree that should be used to perform phylogenetic

diversity analyses (e.g., with

core_diversity_analyses.py).

-p PARAMETER_FP, --parameter_fp=PARAMETER_FP

path to the parameter file, which specifies changes to

the default behavior. See

http://www.qiime.org/documentation/file_formats.html

#qiime-parameters . [if omitted, default values will

be used]

-t TAXONOMY_FP, --taxonomy_fp=TAXONOMY_FP

the taxonomy map [default:

/home/qiime/1.9.0/software/gg_otus-

13_8-release/taxonomy/97_otu_taxonomy.txt]

-s, --assign_taxonomy

Assign taxonomy to each sequence using

assign_taxonomy.py (this will override --taxonomy_fp,

if provided) [default: False]

-f, --force Force overwrite of existing output directory (note:

existing files in output_dir will not be removed)

[default: none]

-w, --print_only Print the commands but don't call them -- useful for

debugging [default: False]

-a, --parallel Run in parallel where available [default: False]

-O JOBS_TO_START, --jobs_to_start=JOBS_TO_START

Number of jobs to start. NOTE: you must also pass -a

to run in parallel, this defines the number of jobs to

be started if and only if -a is passed [default: 200]

--suppress_taxonomy_assignment

skip the taxonomy assignment step, resulting in an OTU

table without taxonomy (this will override

--taxonomy_fp and --assign_taxonomy, if provided)

[default: False]

REQUIRED options:

The following options must be provided under all circumstances.

-i INPUT_FP, --input_fp=INPUT_FP

the input sequences [REQUIRED]

-o OUTPUT_DIR, --output_dir=OUTPUT_DIR

the output directory [REQUIRED]

Comparative samples, beyond pick_closed_otus

Due to the poor performance of the QIIME’s *pick_closed_otus.py* script on OTU assignments for cockatiels, we decided to perform the very same analysis described in previous sections (1) and (2) of this log to process each of the animal species individually.

Briefly:

1. QC filtering
2. OTU clustering
3. OTU taxonomic assignment
4. OTU chimera, and contaminant removal
5. OTU table generation with taxonomy
6. Perform the summarize taxa script for each individual study (animal):

| $for N in `cat list`; do echo "summarize_taxa.py -i $N.biom -o $N.tax.sum"; done |
| --- |

1. Merge the phyla level taxonomic summaries (Level 2) of the previous step by the taxonomic assignment. Until get a tab separated file of the following shape:

|  | kakapo1 | kakapo2 | kakapo3 | ninfa1 | ninfa2 | ninfa3 | … |
| --- | --- | --- | --- | --- | --- | --- | --- |
| No_blast_hit_Other | 0.001604493 | 0.009179575 | 0.002186987 | 0.000684932 | 0.000360227 | 0.000334775 | … |
| None_Other | 0.126754914 | 0.193344808 | 0.086386003 | 0 | 0 | 0 | … |
| Unknown | 0 | 0 | 0 | 0 | 0 | 0 | … |
| Acidobacteria | 0 | 0 | 0 | 2.21E-005 | 2.06E-005 | 0 | … |
| Actinobacteria | 0.016446049 | 0.001147447 | 0 | 0.000198851 | 0.000494025 | 4.65E-005 | … |
| Armatimonadetes | 0 | 0 | 0 | 0 | 0 | 0 | … |
| BHI80-139 | 0 | 0 | 0 | 0 | 0 | 0 | … |
| BRC1 | 0 | 0.000573723 | 0 | 0 | 0 | 0 | … |
| Bacteroidetes | 0 | 0.025243832 | 0.006014215 | 0.000419797 | 0.000535194 | 0.000158088 | … |
| Chloroflexi | 0 | 0.000573723 | 0 | 0 | 0 | 9.30E-006 | … |
| Cyanobacteria | 0.001604493 | 0.016064257 | 0.019682887 | 8.84E-005 | 0.003838989 | 0.001711071 | … |
| Deferribacteres | 0 | 0 | 0 | 0 | 0 | 0 | … |
| Elusimicrobia | 0 | 0 | 0 | 0 | 0 | 0 | … |

1. Use table (step 7) as input in R to elaborate barplots:

| library(ggplot2) library(phyloseq) phylum <- read.table("otu_table_L2.txt", header=T, row.names=1) abund_table <- t(phylum) x <- abund_table[,order(colSums(abund_table), decreasing=T)] for (i in 1:dim(x)[2]){ tmp<-data.frame(row.names=NULL,Sample=rownames(x),Taxa=rep(colnames(x)[i],dim(x)[1]),Value=x[,i]) if(i==1){df<-tmp} else {df<-rbind(df,tmp)} } dat$V1 <- factor(df$Sample, levels=(unique(rownames(x)))) N <- length(df$Taxa) colours <- c("darkorange1", "snow3", "green3", "navy", "greenyellow", "purple", "turquoise4", "skyblue", "violetred", "yellow", "red", "purple4", "turquoise1", "violet", "slateblue4", "olivedrab", "tomato4", "aquamarine") p <- ggplot (df, aes(Sample, Value, fill=Taxa)) + geom_bar(stat="identity") p2 <- p + scale_fill_manual(values=colours[1:(N+1)]) p2 <- p2 +theme_bw() +theme(axis.text.x=element_text(angle=90, hjust=1, vjust=0.5)) p2 <- p2 + xlab("") + ylab("Relative Frequency") ggsave("bar_phylum.pdf") |
| --- |
