# Supplementary material for: Exploring the cockatiel (Nymphicus hollandicus) fecal microbiome, bacterial inhabitants of a worldwide pet
Source: PeerJ. 2016 Dec 22;4:e2837. doi: 10.7717/peerj.2837 (PMC5183021; doi:10.7717/peerj.2837)
Supplement: Table S1 [file peerj-04-2837-s002.pdf]

CatchAll Total Diversity Estimations

Cockatiel 1

Total Number of Observed Species = 6932	Model	Tau	Observed Sp	Estimated Total Sp	SE	Lower CB	Upper CB	GOF0	GOF5
Best Parm Model	ThreeMixedExp	73	6885	152809.1	61766.6	72746.5	330267.3	0.0108	0.529
Parm Model 2a	ThreeMixedExp	71	6884	157616	67864.3	71834	356777.3	0.0122	0.544
Parm Model 2b	ThreeMixedExp	134	6900	101281.6	16211.9	74468.7	138739.5	0	0.014
Parm Model 2c	ThreeMixedExp	64	6882	167600.6	81570.3	69781.3	417667.1	0.0041	0.274
WLRM	LogTransf	29	6848	27174.6	3798.2	20990.2	36079.6		
Parm Max Tau	ThreeMixedExp	34765	6932	50193.1	1899.1	46628.1	54078.3		0
WLRM Max Tau	LogTransf	29	6848	27174.6	3798.2	20990.2	36079.6		
Best Discounted	TwoMixedExp	73	2131	4480.1	1792.4	2775	11051		
Total Number of Observed Species = 6932	Model	Tau	Observed Sp	Estimated Total Sp	SE	Lower CB	Upper CB	CV_rare	
Non-P 1	Chao1	2	6736	32837.4	1264.6	30475	35436.8		
Non-P 2	ACE1	10	6736	76679.4	4640.4	68161.1	86382.7	1.16	
Non-P 3	ACE	10	6736	37869	1367.7	35302.3	40667.8	1.16	

Cockatiel 2

Total Number of Observed Species = 7537	Model	Tau	Observed Sp	Estimated Total Sp	SE	Lower CB	Upper CB	GOF0	GOF5
Best Parm Model	FourMixedExp	273	7517	101814.8	12865.6	79778	130573.9	0.0101	0.686
Parm Model 2a	FourMixedExp	114	7494	118966.3	39602.9	64215.7	226604.8	0.2771	0.798
Parm Model 2b	FourMixedExp	273	7517	101814.8	12865.6	79778	130573.9	0.0101	0.686
Parm Model 2c	FourMixedExp	17	7387	139147.7	38227.9	82878.5	237441.6	0.0157	0.016
WLRM	LogTransf	32	7440	29390.8	4141.3	22660.1	39117.1		
Parm Max Tau	FourMixedExp	32707	7537	64288.2	2771.3	59111.2	69984.9		0
WLRM Max Tau	LogTransf	32	7440	29390.8	4141.3	22660.1	39117.1		
Best Discounted	ThreeMixedExp	273	1548	2450.5	307.2	2022.8	3280.3		
Total Number of Observed Species = 7537	Model	Tau	Observed Sp	Estimated Total Sp	SE	Lower CB	Upper CB	CV_rare	
Non-P 1	Chao1	2	7330	36515.2	1367.7	33956.1	39322.1		
Non-P 2	ACE1	10	7330	87619	4795.7	78757.5	97583	1.19	
Non-P 3	ACE	10	7330	41822.4	1407.7	39172.5	44694.3	1.19	

Cockatiel 3

Total Number of Observed Species = 7129	Model	Tau	Observed Sp	Estimated Total Sp	SE	Lower CB	Upper CB	GOF0	GOF5
Best Parm Model	ThreeMixedExp	80	7080	160647.9	31449	110304.3	235556.4	0.0889	0.129
Parm Model 2a	ThreeMixedExp	77	7078	164668.6	34007.1	110817.6	246487.2	0.0978	0.198
Parm Model 2b	ThreeMixedExp	190	7109	123936.8	13902	99709.5	154504	0	0.012
Parm Model 2c	FourMixedExp	33	7040	289002.4	52568.8	203310.8	412123.8	0.0004	0.289
WLRM	LogTransf	26	7028	32070.2	5965.1	22838.4	46727.2		
Parm Max Tau	ThreeMixedExp	33289	7129	74200.9	3642.1	67433.9	81727.3		0
WLRM Max Tau	LogTransf	26	7028	32070.2	5965.1	22838.4	46727.2		
Best Discounted	TwoMixedExp	80	1475	2448	469.8	1885.1	3889.2		
Total Number of Observed Species = 7129	Model	Tau	Observed Sp	Estimated Total Sp	SE	Lower CB	Upper CB	CV_rare	
Non-P 1	Chao1	2	6932	42354	1829.6	38946.8	46126.2		
Non-P 2	ACE1	10	6932	101694.4	6067.2	90530.7	114352.4	1.24	
Non-P 3	ACE	10	6932	45597.7	1684.1	42436	49042.5	1.24	

CatchAll Diversity Estimations without singletons

Cockatiel 1

Total Number of Observed Species = 879	Model	Tau	Observed Sp	Estimated Total Sp	SE	Lower CB	Upper CB	GOF0	GOF5
Best Parm Model	ThreeMixedExp	1936	876	1092.8	20.4	1056.4	1136.5		0
Parm Model 2a	ThreeMixedExp	1734	875	1093.4	20.5	1056.8	1137.5		0
Parm Model 2b	ThreeMixedExp	2006	877	1092.2	20.3	1056.1	1135.7		0
Parm Model 2c	Poisson	7	631	911.8	6.4	901.5	926.9		0
WLRM	LogTransf	29	795	932.8	13.7	911.9	966.9		
Parm Max Tau	ThreeMixedExp	34765	879	1073.4					0
WLRM Max Tau	LogTransf	29	795	932.8	13.7	911.9	966.9		
Best Discounted	TwoMixedExp	1936	198	207.2	3.8	202.9	219.5		
Total Number of Observed Species = 879	Model	Tau	Observed Sp	Estimated Total Sp	SE	Lower CB	Upper CB	CV_rare	
Non-P 1	Chao1	2	683	978.7	24	941.7	1037.7		
Non-P 2	ACE	10	683	931.6	10.4	914.9	956.1	0.38	
Non-P 3	ACE	10	683	931.6	10.4	914.9	956.1	0.38	

Cockatiel 2

Total Number of Observed Species = 1000	Model	Tau	Observed Sp	Estimated Total Sp	SE	Lower CB	Upper CB	GOF0	GOF5
Best Parm Model	ThreeMixedExp	1051	993	1251.6	21.8	1212.4	1298		0
Parm Model 2a	ThreeMixedExp	1188	995	1250.7	21.6	1211.8	1296.8		0
Parm Model 2b	ThreeMixedExp	897	992	1252.1	21.9	1212.6	1298.8		0
Parm Model 2c	ThreeMixedExp	1073	994	1251.1	21.7	1212.1	1297.4		0
WLRM	LogTransf	32	903	1067.6	13.6	1045.7	1099.9		
Parm Max Tau	ThreeMixedExp	32707	1000	1241.2					0
WLRM Max Tau	LogTransf	32	903	1067.6	13.6	1045.7	1099.9		
Best Discounted	TwoMixedExp	1051	186	197.7	3.3	194.5	209.8		
Total Number of Observed Species = 1000	Model	Tau	Observed Sp	Estimated Total Sp	SE	Lower CB	Upper CB	CV_rare	
Non-P 1	Chao1	2	793	1121.3	25.8	1080.3	1183.3		
Non-P 2	ACE	10	793	1069.3	12.1	1049.3	1097.4	0.4	
Non-P 3	ACE	10	793	1069.3	12.1	1049.3	1097.4	0.4	

Cockatiel 3

Total Number of Observed Species = 991	Model	Tau	Observed Sp	Estimated Total Sp	SE	Lower CB	Upper CB	GOF0	GOF5
Best Parm Model	TwoMixedExp	187	969	1275.9	23.8	1232.9	1326.5	0	0
Parm Model 2a	ThreeMixedExp	671	982	1283	25.1	1237.8	1336.4		0
Parm Model 2b	TwoMixedExp	185	968	1276.5	23.9	1233.4	1327.3	0	0
Parm Model 2c	TwoMixedExp	179	966	1277.9	24	1234.5	1329	0	0
WLRM	LogTransf	13	829	1102.8	30	1057.7	1178.3	0	
Parm Max Tau	TwoMixedExp	33289	991	1196.2	17.6	1164.5	1233.8		0
WLRM Max Tau	LogTransf	26	890	1088.5	18.4	1058.5	1131.8		
Best Discounted	SingleExp	187	167	193.3	3.3	190.2	205.3		
Total Number of Observed Species = 991	Model	Tau	Observed Sp	Estimated Total Sp	SE	Lower CB	Upper CB	CV_rare	
Non-P 1	Chao1	2	794	1140.4	28.6	1094	1207.7		
Non-P 2	ACE	10	794	1094.5	16	1067.6	1130.9	0.47	
Non-P 3	ACE	10	794	1094.5	16	1067.6	1130.9	0.47	

Total Breakaway Diversity

Cockatiel 1

```
    The best estimate of total diversity is 169674
    with std error 1608005
    The model employed was model_1_1
    The function selected was
     f_{x+1}/f_{x} ~ (beta0+beta1*(x-xbar))/(1+alpha1*(x-xbar))
      Coef estimates Coef std errors
beta0      1.21610693      0.22172786
beta1      0.08245387      0.02488437
alpha1     0.02805390      0.03525302
xbar            14.5>
```

Cockatiel 2

```
No breakaway models converged.
    The best estimate of total diversity is 29391
    with std error 4141
    The model employed was the WLRM
```

Cockatiel 3

```
    The best estimate of total diversity is 8502
    with std error 44975
    The model employed was model_2_1
    The function selected was
     f_{x+1}/f_{x} ~ (beta0+beta1*(x-xbar)+beta2*(x-xbar)^2)/(1+alpha1*(x-xbar))
      Coef estimates Coef std errors
beta0     1.064391571     0.298957844
beta1     0.127222455     0.040093932
alpha1    0.077637606     0.022635481
beta2     0.003250292     0.004111814
xbar            13
```

Breakaway diversity without singletons

Cockatiel 1

```
No breakaway models converged.
    The best estimate of total diversity is 933
    with std error 14
    The model employed was the WLRM
```

Cockatiel 2

```
No breakaway models converged.
    The best estimate of total diversity is 1068
    with std error 14
    The model employed was the WLRM
```

Cockatiel 3

```
No breakaway models converged.
    The best estimate of total diversity is 1088
    with std error 18
    The model employed was the WLRM
```
