# Supplementary figures and images for: Exploring the cockatiel (Nymphicus hollandicus) fecal microbiome, bacterial inhabitants of a worldwide pet
Source: PeerJ. 2016 Dec 22;4:e2837. doi: 10.7717/peerj.2837 (PMC5183021; doi:10.7717/peerj.2837)

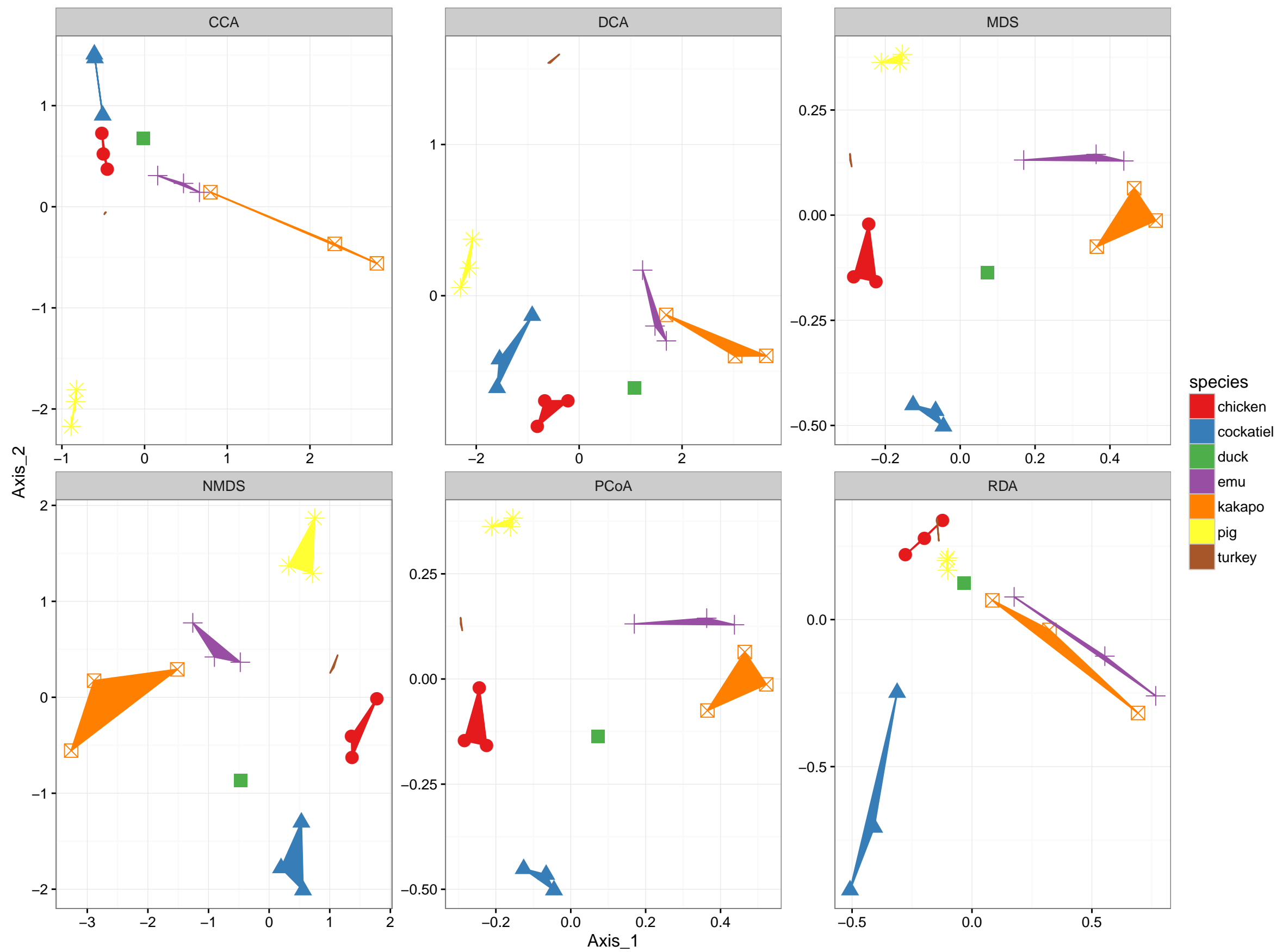

Supplement: Figure S1 [file peerj-04-2837-s003.pdf]

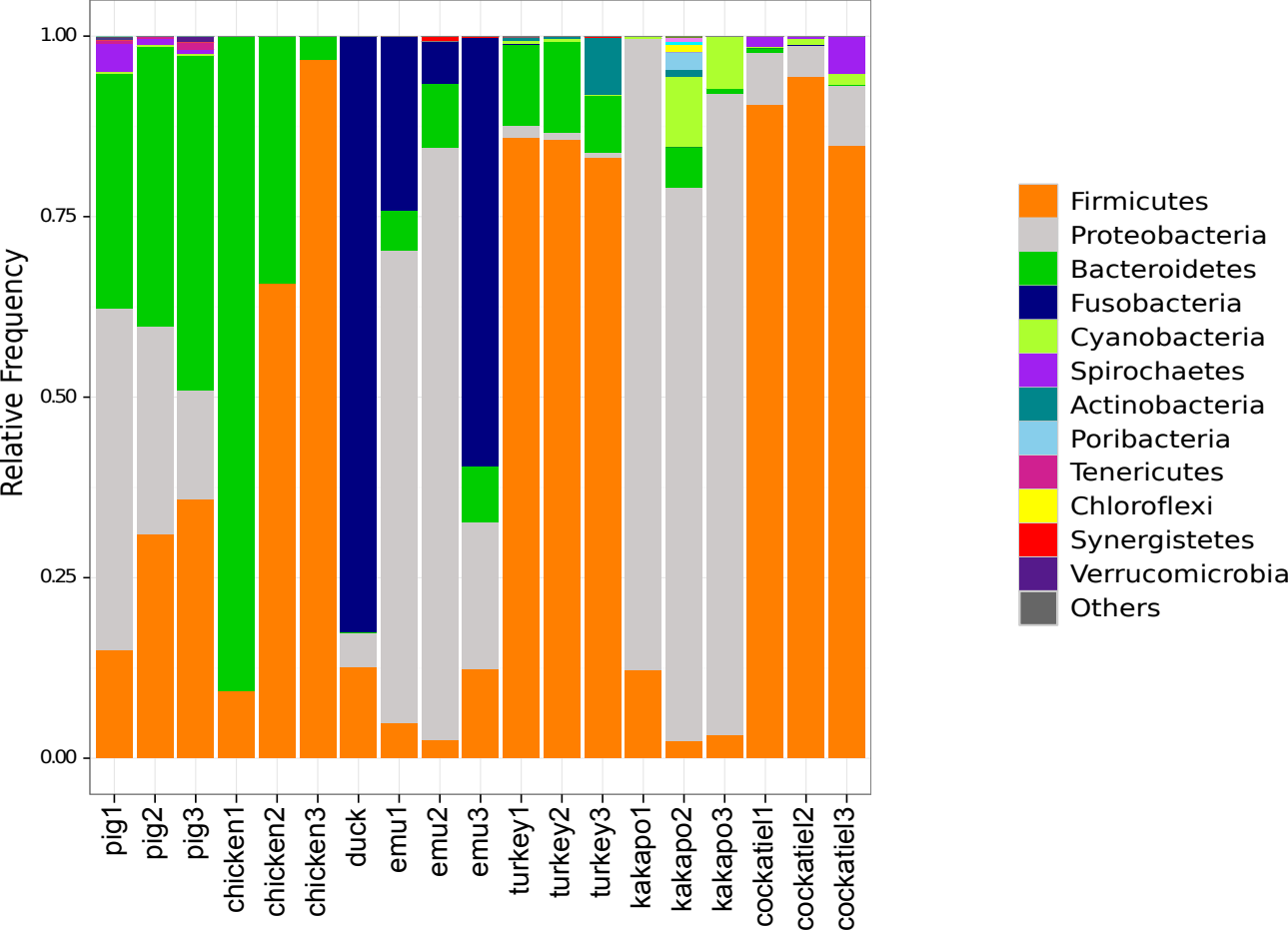

Supplement: Figure S2 [file peerj-04-2837-s004.pdf]

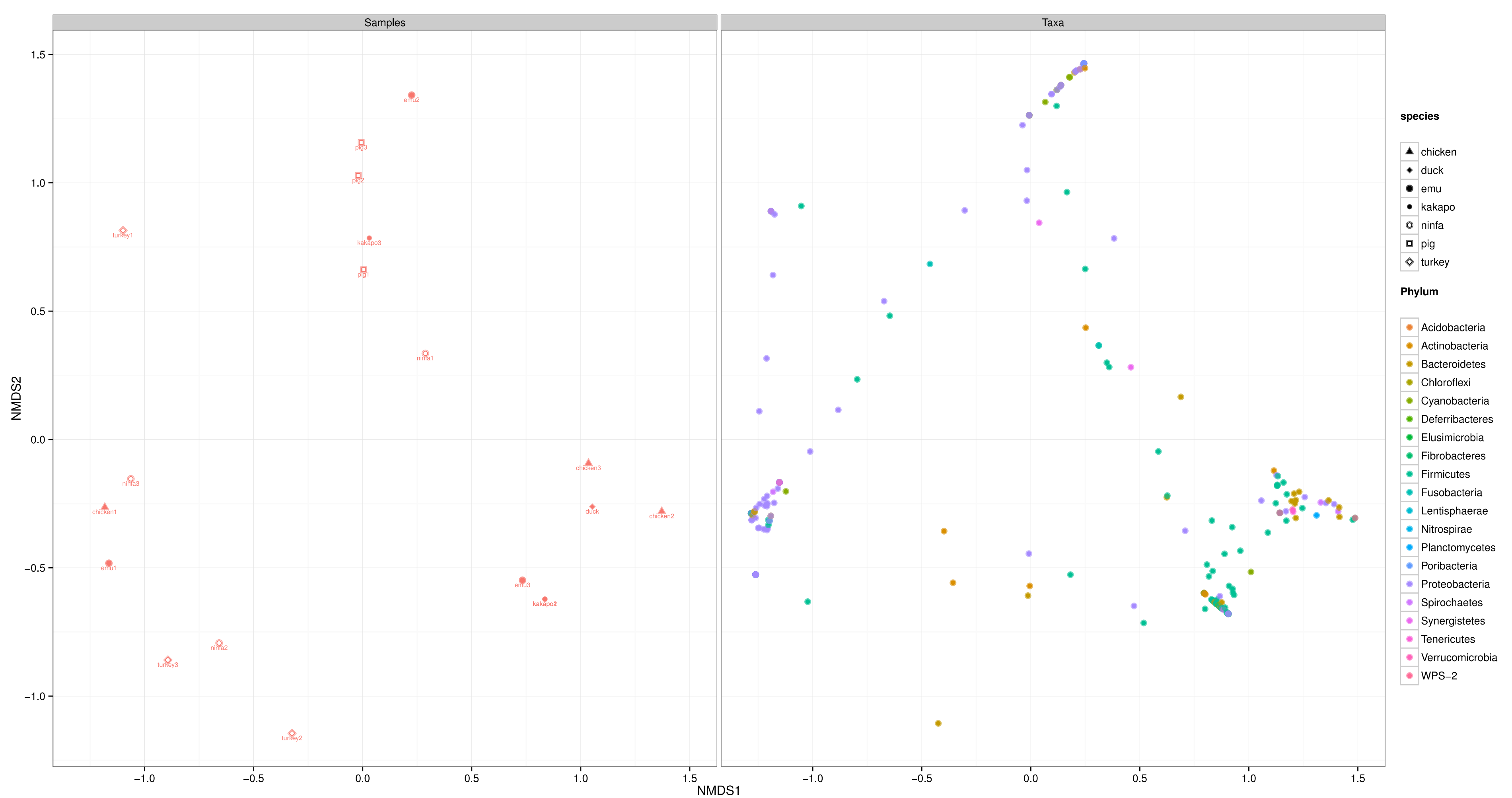

Supplement: Figure S3 [file peerj-04-2837-s005.pdf]
